# Enhancing antimicrobial stewardship program: impact of clinical pharmacist-driven feedback in the absence of infectious diseases physicians—a multicenter quasi-experimental study

**DOI:** 10.1017/ash.2025.10088

**Published:** 2025-09-03

**Authors:** Anup R. Warrier, Nalluri Tejaswini, Hafeedha Kallarakkal, Soumya Sagar

**Affiliations:** 1 Department of Infectious Diseases, Aster Medcity, Kochi, Kerala, India; 2 Aster Ramesh Hospitals, Vijayawada, Andhra Pradesh, India; 3 Aster MIMS Hospital, Calicut, Kerala, India; 4 Aster RV Hospital, Banglore, Karnataka, India

## Abstract

**Objective:**

To evaluate the impact of clinical pharmacist-driven feedback on Antimicrobial Stewardship Program (AMSP) in the absence of infectious disease physicians across three different geographic locations.

**Design:**

Multicenter quasi-experimental study.

**Setting:**

Three private tertiary referral centers in different geographical locations in India.

**Participants:**

All consecutive prescriptions with restricted antibiotics for inpatients during the study period.

**Intervention:**

This study was conducted over 15 months from June 2022 to May 2023. The impact of mentoring clinical pharmacists by infectious disease physicians, enhancing their communication abilities for providing proactive feedback, and the impact on prescription practice were measured in terms of new prescriptions of restricted antibiotics, compliance to clinical pharmacist advice, and the duration of restricted antibiotic therapy usage, measured in terms of days of therapy (DOT) of restricted antibiotics. Gross mortality was reviewed as a balancing measure, and dose/dosing errors were considered as a secondary outcome. Data were captured in Microsoft Excel and analyzed using the SPSS software.

**Results:**

Clinical pharmacist-led antimicrobial stewardship interventions were found to have a significant impact on decreasing antibiotic prescriptions, increasing healthcare organization policy compliance, and decreasing DOT for restricted antibiotics. Culture sampling, acceptance of antimicrobial stewardship advice, dosing errors, or mortality rates were not statistically significantly related to the other study parameters.

**Conclusion:**

Clinical pharmacist-driven AMSP can be effectively implemented irrespective of the cultural and geographical setting due to their ability to improve prescription practices.

## Introduction

Antimicrobial resistance (AMR) is a threat to public health due to the emergence of newer resistant bacteria that outpace the development of newer antibiotics. According to the literature, 50% of the antibiotics are either unnecessarily or inappropriately prescribed in India which may lead to the development of AMR.^1,2^ The treatment of common infections becomes more challenging, leading to increased length of hospital stay, healthcare expenses, and even mortality rates.^3^ Hence, multifaceted strategies such as improving infection prevention and control, implementing Antimicrobial Stewardship Programs (AMSP), enhancing surveillance, and developing new antimicrobial agents should be implemented to address this issue.^4^

AMSP is defined as a coordinated effort to promote the appropriate use of antimicrobials to improve patient outcomes, reduce resistance, and decrease the spread of resistant infections. Successful implementation of an AMSP requires multiple stakeholders, of which the infectious disease physician is often at the center. This is because key aspects of implementation, such as the right selection of antimicrobials for a clinical syndrome and influencing the prescription behavior of other clinicians, often require a clinical leader. Trained and experienced infectious disease practitioners are very few compared to their demand. However, the development of a hospital antibiotic policy based on best practices, review of the use of “restricted antimicrobials” based on predefined criteria, and monitoring of the proposed duration of antimicrobial use can be successfully performed by trained clinical pharmacists. This study aimed to evaluate the impact of clinical pharmacist-driven feedback on AMSP in the absence of infectious disease physicians across three different geographic locations.

## Methodology

A quasi-experimental study was conducted in hospitals of various sizes (600 beds, 150 beds, and 250 beds) in three different geographies (Kerala, Karnataka, and Andhra Pradesh) in India over 15 months from May 2022 to July 2023. All three hospitals were part of the same system in various states of India. As the study was a quality improvement initiative, Institutional Ethics Committee approval was not deemed necessary before the initiation of the study. All consecutive prescriptions with restricted antibiotics for inpatients during the study period were included and all other prescriptions were excluded.

### Intervention description

In all three hospitals, one clinical microbiologist was available for laboratory services, and there was no established AMSP. However, all of them had published hospital antibiotic policies according to the National Accreditation Board for Hospitals and Healthcare Providers accreditation requirements. A clinical pharmacist was recruited in each of the hospitals for the program, mentored by the Project Champion (Group Consultant for Infectious Diseases—MBBS, DNB general medicine, Fellowship in ID, 18 yr clinical practice). After the recruitment, the pharmacist was trained during rounds and lectures by the champion, regarding the antibiotic policy and syndrome based recognition of infectious diseases following simple criteria.

### Audit period

During the first three months of the project (May 2022 to July 2022), baseline adherence to the published antibiotic policy, dose/dosing errors in antimicrobial prescriptions, and the consumption of restricted groups of antimicrobials (according to WHO AWaRe classification)^5^ in terms of “Days of Therapy” (DOT) was captured. There was no feedback or any interaction between the clinical pharmacist and the prescriber or treatment team.

### Audit-and-Feedback intervention period

In intervention initiation phase in the next six months (August 2022–January 2023), feedback was provided to the clinical team, admitting doctors, and prescribers at individual level on the same day as prescription review, in person or via phone call, to ensure compliance with the hospital antibiotic policy when prescribing restricted antimicrobials. Antimicrobial indication was not documented by the prescriber, but assessed using a predefined criteria by the pharmacist. Adherence to policy, dose/dosing errors, and DOT of the restricted antimicrobials were recorded throughout. Weekly data review was done by the champion to constantly give feedback to the pharmacist on syndrome based diagnosis. The aggregate of these parameters at the end of six months was compiled and presented to the prescribers by the Champion, which included common deviations and possible improvements. Suggestions to improve adherence, any changes required in the hospital’s antibiotic policy, and measures to decrease errors were sought.

After the intervention, during the intervention consolidation phase in the next six months (February 2023–July 2023), the intervention was continued with real-time, prospective feedback on prescriptions by clinical pharmacists. The aggregate of the defined indicators was compiled again at the end of six months. All the data were collected by the clinical pharmacist in real time and followed up for any changes in the prescriptions

### Outcome measured

The primary outcome of the study was DOT for restricted antibiotics. Secondary outcomes include compliance to the hospital antibiotic policy, acceptance of antimicrobial stewardship advice, dosing errors, and gross mortality.

### Statistical analysis

For categorical data, the χ^2^ test or Fisher’s exact test was used to compare the proportions between different time points. For continuous variables, t-tests or ANOVA were used to compare the means. In this study, we used a 95% confidence interval, and the results were considered statistically significant if the *P* value was less than .05. The outcomes of each study period were compared using Poisson rate ratio. Microsoft Excel was used to collect all the data, and it was then analyzed using SPSS version 20.00.

## Results

The key metrics that were reviewed for the study were—the number of new prescriptions for restricted antimicrobials during the study phases (representing the number of new starts), the “Days of Therapy” for restricted antimicrobials (representing total use), the percentage of patients for whom appropriate cultures were sent before initiation of restricted antimicrobials, percentage of patients in whom the antibiotic selection at initiation was according to the hospital policy, percentage of patients in whom the clinical pharmacist suggestion for a review of the antibiotic choice was accepted, instances of dose and dosing errors in the primary prescription and finally, the gross mortality at 30 days in those patients on restricted antimicrobials. Details are presented in Table 1.

**Table 1.** Comparison of prescription pattern at baseline and postintervention

Key performance indicators	Baseline (3 months)	Intervention initiated (6 months)	Intervention consolidated (6 months)	Test statistic (Sig.)
Number of prescriptions for restricted antibiotics	1,335	2,455	2,376	218.627^a^ (*P* < .001*)
% of patients wherein appropriate samples were collected for cultures	81.60%	92%	93%	4.333^a^ (*P* = .114)
Compliance with antibiotic selection according to healthcare organization policy	53.70%	69.70%	76%	59.868^a^ (*P* < .001*)
Acceptance of antimicrobial stewardship advice for review of antibiotics regimen	65%	70.60%	70%	1.350^a^ (*P* = .509)
Dose and/or Dosing error (frequency)	2.35%	1%	1.10%	4.650^a^ (*P* = .097)
Days of therapy for restricted antibiotics	415/1,000 IP days	404/1,000 IP days	391/1,000 IP days	27.153^b^ (*P* < .001*)
Gross mortality at 30 days	17.20%	18%	17.10%	.124^b^ (*P* = .885)

a Fisher’s exact test.

b Anova test.

*P value <.05 shows significance.

Different key performance indicators are evaluated in the table to determine the impact of a clinical pharmacist-driven antimicrobial stewardship intervention. The number of prescriptions for restricted antibiotics during the baseline audit period was 1,335 (approximately 445 per month). However, after six months of intervention, the number of antibiotic prescriptions audited accounted for 2,376 (approximately 396 prescriptions per month), showing a reduction in the number of antibiotic prescriptions after the intervention (*P* < .001).

Initially, 81.6% of patients had appropriate samples collected for cultures. This was increased to 92% at the time of intervention. There was a slight increase to 93% after six months when the intervention was consolidated. However, this increase was not statistically significant (*P* = .114). Considering policy compliance, only 53.7% of prescriptions showed compliance with healthcare organization policy, the most common deviation from the policy being empirical choice of antibiotics for LRTI and duration of antibiotic use. There was a statistically significant improvement in compliance with healthcare organization policies from 53.70% to 69.70% (during intervention) and then to 76% after intervention (*P* < .001). Acceptance of antimicrobial stewardship advice showed a slight, nonsignificant increase from 65% to 70.60% and 70% (*P* = .509), and the reduction in dose and/or dosing errors from 2.35% to 1% and 1.10% was also not statistically significant (*P* = .097).

During the initial audit period of three months, DOT was approximately 415 DOT per 1,000 IP days. After the intervention period of 6 months, the total DOT increased 404 DOT per 1,000 IP days. When adjusted for a longer period (six months), the actual DOT per month decreased to 391 DOT per 1,000 IP days. The statistical significance of this comparison was calculated using poisson rate ratio at 95% confidence interval, which was 0.97 on comparing baseline data and interventional data, and 0.94 on comparing baseline data and consolidated data. The latter was 5.8% lower than the former, and was found to be statistically significant. Hospital wise stratification of the outcome was not done, which can be taken as a confounder of the study.

## Discussion

This multicenter interventional study evaluated the impact of clinical pharmacist-driven feedback on AMSP in the absence of infectious disease physicians. Our study was conducted at three different geographic locations. The study showed that the clinical pharmacist-led intervention could significantly reduce antibiotic prescriptions, improve compliance with HCO policy, and reduce DOT for restricted antibiotics. Consistent and prospective feedback about adherence to hospital antibiotic policies coupled with alerts and inputs on the microbiological results by a Clinical Pharmacist can improve prescription practices among clinicians.

A multicenter point prevalence study conducted by the NCDC for the year 2021–22 highlighted the magnitude of inappropriate antimicrobial prescriptions in India, with a high prevalence of antibiotic usage of 71.9%.^6^ Against this background, we looked at studies that incorporated clinical pharmacist-led interventions as a core strategy for improving prescription practices. A Cochrane review by Davey et al emphasized the importance of interventions in improving antibiotic prescribing practices for hospital inpatients.^7^ Enabling interventions with feedback was an effective strategy in this review. Hence, this was the core intervention considered in this study.

The possible contributions of clinical pharmacists in developing countries to the promotion of AMSP have been published in a narrative review by Sakeena et al.^8^ The review highlighted the valuable contributions made by clinical pharmacists in reducing the development and dissemination of AMR. Delivering real-time interventions that could reduce inappropriate antibiotic use and collaborating with other healthcare professionals help to optimize antibiotic use. This was achieved by providing up-to-date information on antibiotics, adverse drug reactions, and monitoring of antibiotic use.

The role of clinical pharmacists focusing on practices in Asia was comprehensively covered in a systematic review published by Jantarathaneewat et al,^4^ and in Sub-Saharan Africa by Otieno et al in 2022.^9^ According to their report, clinical pharmacists play a crucial role in antimicrobial stewardship initiatives in Asia, and their contributions were significant in enhancing the use of antimicrobials and improving patient outcomes. Moreover, their role goes beyond traditional drug-related activities, which include prescribing and supervising antimicrobials, as well as educational initiatives and policy formulation. Clinical pharmacists also play a vital role in multidisciplinary healthcare teams, working with doctors, nurses, microbiologists, and other medical professionals to improve patient outcomes. They aim to optimize antimicrobial therapy by ensuring proper drug selection, dosage, treatment duration, and patient monitoring, thus helps in reducing the potential for AMR and unwanted medication side effects. Their study also underscores the importance of educational and training initiatives designed to provide clinical pharmacists with the necessary expertise and capabilities for successful involvement in AMSP.

We examined the utilization of this emerging resource in healthcare delivery in Indian settings to improve antimicrobial stewardship. Published studies that examined clinical pharmacist-led interventions in India are available as precedence. An Indian study by Dawaiwala et al showed that interventions by clinical pharmacists could enhance antimicrobial therapy and decrease the daily defined doses of restricted antimicrobials.^10^ Kuruvilla et al evaluated the impact of the intervention by clinical pharmacists in promoting the rational use of antibiotics by interacting with surgeons in the surgical ward, and reported that they have a crucial role in ensuring the appropriateness of the antibiotics used by closely monitoring prescriptions in a real-time manner.^11^ Our study also reported a decrease in the number of prescriptions, an increase in adherence to healthcare organization policy, and a decrease in DOT for restricted antibiotics. Another study by Namboothiri et al also emphasized that clinical pharmacist-driven AMSP could efficiently bring lasting change in low- and middle-income countries, such as India, where the shortage of infectious disease physicians is a major barrier to driving change.^12^

Cantudo-Cuenca et al highlighted the adaptability of pharmacist-led AMSP in small hospitals without infectious diseases physicians. Despite resource constraints, pharmacist-led interventions were effective in promoting AMSP and in reducing the risk of AMR.^13^ Findings by Dawaiwala et al.^10^ corroborated the results of our study, demonstrating the impact of clinical pharmacist interventions on antimicrobial stewardship in Indian tertiary care hospitals. By implementing pharmacist-led interventions, such as prospective feedback and adherence to hospital antibiotic policies, improvements in prescription practices, and reductions in antimicrobial consumption were observed.

Otieno et al reported that to ensure the successful implementation of pharmacist-led AMSP in Sub-Saharan hospitals, it is crucial to provide training for pharmacists on antimicrobial stewardship interventions, foster collaboration with relevant healthcare stakeholders, mobilize resources, and continuously improve the quality of the program.^9^ Kuruvilla et al^11^ showed that clinical pharmacists play a crucial role in optimizing antibiotic use by monitoring prescriptions for indication, dose, frequency, route, and duration. Their timely suggestions can reduce costs, hospital stays, and adverse drug reactions, ultimately helping to combat antibiotic resistance. Literature also showed the importance of establishing AMSP in hospitals with pharmacists to promote judicious antimicrobial use, which will help developing countries combat AMR.^8^ Thus, the current study aligns with the existing literature on AMSP and underscores the critical role of clinical pharmacists in optimizing antimicrobial use, especially in a developing country like India. The novelty of our study is that we were able to implement this intervention in diverse healthcare settings in different geographic regions, with uniform results. Integrating clinical pharmacists into AMSP is essential for promoting judicious antimicrobial use and combating AMR.

## References

[1] Sulis G , Daniels B , Kwan A , et al. Antibiotic overuse in the primary health care setting: a secondary data analysis of standardised patient studies from India, China and Kenya. BMJ Glob Health 2020;5:1–12. 10.1136/bmjgh-2020-003393 PMC7493125 32938614

[2] Holmes AH , Moore LSP , Sundsfjord A , et al. Understanding the mechanisms and drivers of antimicrobial resistance. Lancet 2016;387:176–187. 26603922 10.1016/S0140-6736(15)00473-0

[3] Martens E , Demain AL. The antibiotic resistance crisis, with a focus on the United States. J Antibiot 2017;70:520–526. 10.1038/ja.2017.30 28246379

[4] Jantarathaneewat K , Camins B , Apisarnthanarak A. The role of the clinical pharmacist in antimicrobial stewardship in Asia: a review. ASHE 2022;2:176. 10.1017/ash.2022.310 PMC9641507 36386007

[5] 2021 AWaRe classification [Internet]. [cited 2024 May 11]. https://www.who.int/publications-detail-redirect/2021-aware-classification

[6] Report of First Multicentric Point Prevalence Survey of Antibiotic Use at 20 NAC-NET Sites [Internet]. National Centre for Disease Control (NCDC). [cited 2024 May 11]. https://ncdc.mohfw.gov.in/reports/

[7] Davey P , Marwick CA , Scott CL , et al. Interventions to improve antibiotic prescribing practices for hospital inpatients.. In: Cochrane Database of Systematic Reviews [Internet]; 2017, 2017 Feb 9 [cited 2024 May 10]. https://doi.wiley.com/10.1002/14651858.CD003543.pub4 10.1002/14651858.CD003543.pub4 PMC6464541 28178770

[8] Sakeena MHF , Bennett AA , McLachlan AJ. Enhancing pharmacists’ role in developing countries to overcome the challenge of antimicrobial resistance: a narrative review. Antimicrob Resist Infect Control 2018;7:63. 29744044 10.1186/s13756-018-0351-z PMC5930749

[9] Otieno PA , Campbell S , Maley S , Obinju Arunga T , Otieno Okumu M. A systematic review of pharmacist-led antimicrobial stewardship programs in sub-saharan Africa. Int J Clin Pract 2022;2022:3639943. 36311485 10.1155/2022/3639943 PMC9584722

[10] Dawaiwala I , Raut S , Fuse M , et al. Antimicrobial stewardship and clinical pharmacist interventions in an Indian tertiary care hospital. J Am Coll Clin Pharm 2024;7:46–54.

[11] Kuruvilla AV , Madhan R , Chandagal Puttaswamy M. Clinical pharmacist-initiated assessment and amelioration of appropriate antibiotic use in surgical units at a South Indian tertiary care hospital - a handshake approach. J Infect Dev Ctries 2023;17:66–72. 36795926 10.3855/jidc.17032

[12] Nampoothiri V , Sudhir AS , Joseph MV , et al. Mapping the implementation of a clinical pharmacist-driven antimicrobial stewardship programme at a tertiary care centre in south India. Antibiotics 2021;10:220. 33672095 10.3390/antibiotics10020220 PMC7926893

[13] Cantudo-Cuenca MR , Jiménez-Morales A , Martínez-de la Plata JE. Pharmacist-led antimicrobial stewardship programme in a small hospital without infectious diseases physicians. Sci Rep 2022;12:9501. 35680946 10.1038/s41598-022-13246-6 PMC9184508

